# Logic Programming with Post-Quantum Cryptographic Primitives for Smart Contract on Quantum-Secured Blockchain

**DOI:** 10.3390/e23091120

**Published:** 2021-08-28

**Authors:** Xin Sun, Piotr Kulicki, Mirek Sopek

**Affiliations:** 1 Department of the Foundations of Computer Science, The John Paul Catholic University of Lublin, 20-502 Lublin, Poland; xin.sun.logic@gmail.com; 2 MakoLab SA, 91-062 Lodz, Poland; sopek@makolab.com

**Keywords:** logic programming, quantum blockchain, smart contract

## Abstract

This paper investigates the usage of logic and logic programming in the design of smart contracts. Our starting point is the logic-based programming language for smart contracts used in a recently proposed framework of quantum-secured blockchain, called Logicontract (LC). We then extend the logic used in LC by answer set programming (ASP), a modern approach to declarative logic programming. Using ASP enables us to write various interesting smart contracts, such as conditional payment, commitment, multi-party lottery and legal service. A striking feature of our ASP implementation proposal is that it involves post-quantum cryptographic primitives, such as the lattice-based public key encryption and signature. The adoption of the post-quantum cryptographic signature overcomes a specific limitation of LC in which the unconditionally secure signature, despite its strength, offers limited protection for users of the same node.

A blockchain is a distributed, transparent and append-only chain of cryptographically linked units of data (blocks) stored in a large decentralized network. Due to the mechanisms of introducing new data based on consensus, blockchain can be used by peers who do not trust each other. The data entries can be considered transactions, so a blockchain can be treated as a ledger. Peers in charge of updating the ledger (called often miners) have separated, identical copies of the ledger. This fact makes the system distributed and is crucial for its safety. Recently Bitcoin [1] and other cryptocurrencies made the blockchain technology widely known. Another important and still not fully utilized way of using blockchain is the implementation of smart contracts [2] on their basis. Smart contract is a piece of software that implements an agreement between parties in such a way that terms of the contracts are enforced automatically. Due to registering them in a blockchain, smart contracts are irrefutable, which makes them appropriate for mutually distrusting peers. The main advantage is that a trusted third party is not needed for the affirmation and enforcement of contracts.

The advantages of smart contracts reside in the fact that existing blockchain platforms provide an infrastructure for them. In most cases, procedural languages are supported for smart contracts. However, logic-based languages for smart contracts seem to provide some advantages over the procedural approach [3,4]. The main ones are the following:

- Logical programs in general and smart contracts among them are better suited for formal verification than procedural programs. In the case of procedural programs, a usual way to proceed is to construct a formal calculus with rigorous semantics and express a program as a set of expressions of that calculus [5,6]. Logic is formal calculus itself, so there is no need for translation to other systems, and the verification is easier.
- Logical contracts are usually more compact. In contrast to their procedural counterparts, they are limited only to what has to be done, without specifying how to achieve it.
- Expressing contracts in a logical language is less error prone [7], as they are much closer to the user-friendly specifications than the procedural programming languages.

This paper provides investigations on the usage of logic and logic programming in the design of smart contracts. Our starting point is the logic-based programming language for smart contracts used in the recently proposed framework of Logicontract (LC) [8]. We then extend the logic used in LC by answer set programming (ASP), a modern approach to truly declarative logic programming. Using answer set programming enables us to write various interesting smart contracts. Moreover, due to the well-defined and rigorous syntax and semantics, the contracts written in the ASP language are easier to understand and formally verify.

We assume that the underlying blockchain is a quantum-secured permissioned blockchain, such as LC. More specifically, we assume the existence of a quantum communication network. In this network, every node is a classical computer. It would be better if nodes were quantum computers. In fact, some interesting problems, such as voting, lotteries and auctions, can be solved in an unconditionally secure manner on a blockchain with quantum computers as nodes [9,10]. However, there are still plenty of interesting problems that can be solved on quantum-secured blockchain with classical computers. Nodes are connected by both classical and quantum channels such that unconditionally secure keys between each pair of nodes can be successfully established. Nodes are also the participants (sender or receiver) of transactions. Every transaction is signed by its sender, using a quantum key distribution-based signature scheme. Such a signature is unconditionally secure. Every node maintains a record of all transactions. The consensus algorithm of the blockchain ensures that different nodes have an identical record of transactions.

So far, in LC, there is only unconditionally secure signature (USS), but no public-key signature. While USS is good at protecting messages communicated between different nodes, it seems difficult—at least inconvenient—to protect different users on the same node. To overcome this limitation of USS, we embed post-quantum cryptographic primitives, such as lattice-based public-key signature [11,12,13], into ASP. This treatment allows a user of a node to identify himself from other users by his unique public key. The post-quantum cryptographic primitive makes our logical language even more powerful than ordinary ASP. Our approach is also practically feasible, as witnessed by the very recent work of Wang et al. [14], which experimentally demonstrated the efficiency of the quantum communication network with identity authenticated by post-quantum cryptography.

The structure of this paper is the following. In Section 1, we review the existing work on the programming language and formal models of smart contracts, with special interest in the programming language of LC. We then develop our update of LC in Section 2. Various examples are presented in this section to demonstrate the usage of our logical contract. We conclude this paper with future work perspectives in Section 3.

## 1. Background and Related Work: Programming Language and Formal Models of Bitcoin-like Smart Contract

In this section, we briefly review the existing work on the programming languages and formal models of Bitcoin-like smart contracts. The work on the programming languages and formal models of Ethereum-like smart contracts are also relevant to our paper. However, due to the limitation of space, we will not review them. The interested reader may find a survey of these works by Rouhani [15].

### 1.1. Timed Automata

Andrychowicz et al. [16] proposed a framework for modeling Bitcoin contracts using the timed automata (TA) in the UPPAAL model checker [17]. Their key idea is to use TA to model the behavior of each participant in a contract. The whole system is then modeled as the network constructed by composing these TAs, plus a TA that models the Bitcoin network. The security of the smart contract is then verified in UPPAAL automatically, finding and correcting some subtle errors that are difficult to discover by the manual analysis.

### 1.2. Simplicity

Simplicity [18] is an alternative language for Bitcoin scripts. It is a typed, combinator-based, functional language without loops and recursion. For a formal verification of Simplicity programs, denotational semantics in Coq, a popular, general-purpose software proof assistant, is defined. An abstract machine constituting operational semantics for Simplicity is also provided. It is possible to statically estimate the resources (e.g., memory) required to execute contracts written in Simplicity. In Valliappan et al. [19], the authors connected Simplicity’s primitives with a categorical model. This lifts the language to a more abstract level allowing for extending it by category theory models of computations.

### 1.3. BitML

Bartoletti and Zunino [20] expressed Bitcoin contracts in BitML—a simple process calculus. Contracts are three-phase processes:

1. Participants broadcast a contract advertisement, which specifies the content of the contract and its preconditions (e.g., depositing a certain amount of bitcoins).
2. Participants accept the contract and fulfill all the required preconditions. When all the needed participants commit to the contract, the contract is stipulated and can be executed.
3. Executing the contract eventually results in a transfer of the bitcoins deposited by the participants, according to the logic defined by the contract.

### 1.4. Logical Contract

The logical contract project http://logicalcontracts.com/ (accessed on 22 August 2021) developed a logical representation of a legal document that is close to natural, human language and, at the same time, executable by computer. It can be used for the following:

- Monitor compliance of the parties to a contract;
- Enforce compliance, by automatically performing actions to fulfill obligations, and/or by issuing warnings and remedial actions to respond to violations of obligations;
- Explore logical consequences of hypothetical scenarios;
- Query and update the Ethereum blockchain.

The theoretic foundation of the logical contract project is a LPS (Logic-based Production System), which is a general-purpose computer language, developed by Kowalski, Sadri and Calejo [21,22,23].

### 1.5. Probabilistic Logic Programs for Blockchain and Smart Contracts

Azzolini et al. [24,25,26] applied probabilistic logic programs to model and analyze the safely and effectiveness of blockchain systems. They presented a method to translate smart contracts into probabilistic logic programs that can be used to analyze the expected values of several smart contract’s utility parameters and obtain a quantitative idea on how smart contracts variables changes over time. They have used this method to study several real smart contracts deployed on the Ethereum blockchain.

### 1.6. Logicontract

The programming language for smart contracts on LC [8] is based on the script language of Bitcoin [5,20,27]. The definition of a formula of the language is as follows:

e::=x∣k∣e+e∣Hash(e)

ϕ::=e=e∣e>e∣Odd(e)∣After(e)∣¬ϕ∣ϕ∧ϕ

where ϕ is a formula, *e* is an arithmetic expression, *x* is a variable ranging over natural numbers, k∈N is a constant natural number, Hash is a collision-resistant hash function on natural numbers, and Odd(e) means that *e* is an odd number. LC uses a global clock. After(e) means that the current time by the clock is later than *e*. Propositional operators of negation and conjunction are represented in a usual way by the symbols ¬ and ∧, respectively. The formal definition of the transaction on LC is as follows.

**Definition** **1**  (transaction [8]). *A transaction T is a tuple (send,rece,sour,cert, prot) with the following:*

- *T is the name of the transaction.*
- *send stands for the sender of the transaction.*
- *rece stands for the set of ordered pairs and each of the pairs consists of a potential receiver of this transaction and the amount of the currency that the receiver will receive, i.e., $rece=\{\left(r_{1},a_{1}\right),\ldots ,\left(r_{m},a_{m}\right)\}$.*
- *sour stands for the source of the transaction, which is a list of names of transactions $\left(T_{1},\ldots ,T_{n}\right)$ that are redeemed by T.*
- *prot stands for the protection, which is a list of ordered pairs in which each pair consists of a receiver from rece and a formula that has to be fulfilled by the receiver to redeem the transaction, i.e., $prot=\{\left(r_{1},\phi_{1}\right),\ldots ,\left(r_{m},\phi_{m}\right)\}$. In order to redeem T, $r_{i}$ has to provide $\phi_{i}$ as the certification of a following transaction.*
- *cert stands for the certification, which is a list of ordered pairs consisting of names of transactions and valuation functions that are supposed to satisfy protections of source transactions, i.e., $cert=\{\left(T_{1},V_{1}\right),\ldots ,\left(T_{n},V_{n}\right)\}$. Valuation functions map variables to natural numbers. The list must provide a valuation function for each source transaction.*

A transaction *T* redeems source transactions if and only if the following holds:

1. The sender of *T* is one of the receivers in each of its source transactions.
2. The certification of *T* evaluates correctly the protections of all sources.
3. None of the sources has been already redeemed.

A transaction *T* is redeemed if one of its receivers has redeemed it.

Let us now see how the process of redeeming of one transaction by another on examples.

**Figure 1:**
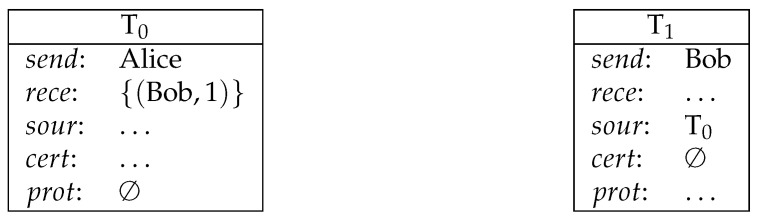
Direct payment.

**Example** **1**  (direct payment). *Bob receives the amount of 1 coin from Alice (see Figure 1).*

**Figure 2:**
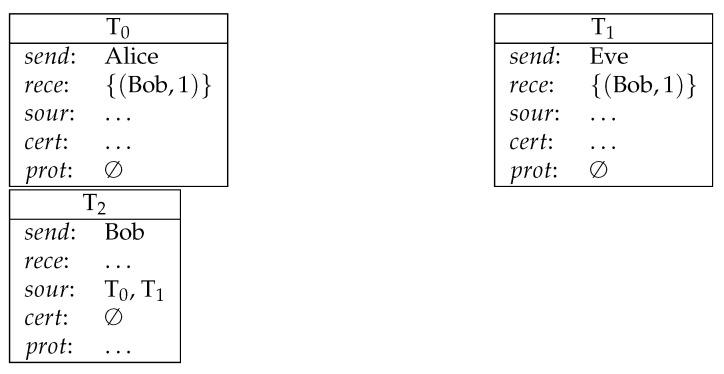
Payment from multiple sources.

**Example** **2**  (payment from multiple sources). *Bob receives payments from Alice and from Eve (1 coin from each) (see Figure 2).*

**Figure 3:**
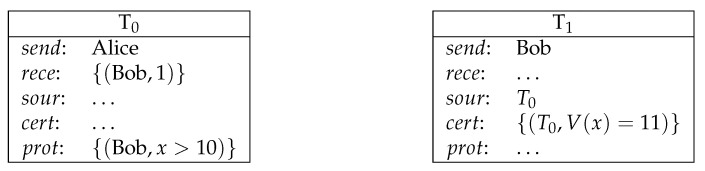
Conditional payment.

**Example** **3**  (conditional payment). *Bob receives the amount of 1 coin from Alice if Bob presents a number larger than 10 as the value of variable x (see Figure 3).*

**Figure 4:**
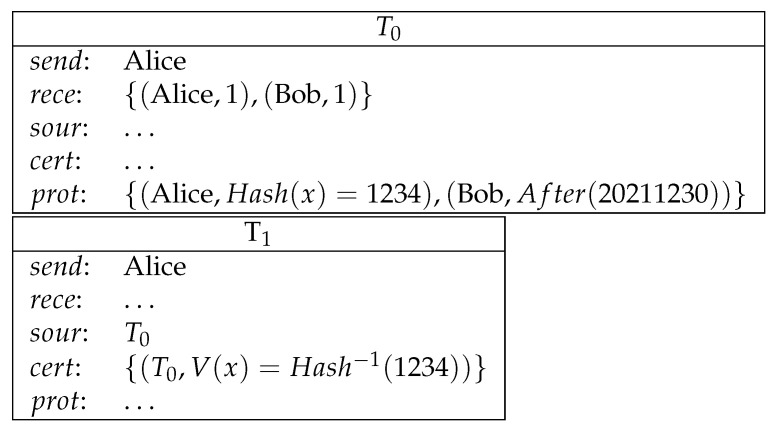
Commitment.

**Example** **4**  (commitment). *Alice commits a secret number x to Bob (the hash value of x is in the example 1234) and makes a deposit (1 coin). If she reveals this secret before a certain time (20211230 in the example), then she redeems the deposit. Otherwise Bob redeems the deposit after the agreed upon time (see Figure 4).*

## 2. Logic Programming for Smart Contracts

The logic used to specify the protection in LC has strong limits on functions and predicates. It can be straightforwardly generalized to involve more expressive logical formulas from logic programming. More specifically, we will use answer set programming [28,29,30], a modern approach to logic programming, as our underlying logic. In the following, we will first review classical logic programming, then review the answer set programming as an extension of classical logic programming.

### 2.1. Classical Logic Programming

Now, we give a formal definition of notions, such as formula, valuation, satisfactions and entailment. All these notions can be found in the literature of logic programming [31,32]. Let X,Y,Z,… stand for variables, a,b,c,… for constants p,q,… for predicate symbols and f,g,h,… for function symbols. Constants can be treated as special cases of functions that have no arguments. A language L of logic programs is determined by the set of its predicates and functions (and constants).

A term is defined inductively as follows: any variable and any constant is a term, and if *f* is an *n*-ary function symbol and $t_{1},\ldots ,t_{n}$ are terms, then $f(t_{1},\ldots ,t_{n})$ is a term. If no variable occurs in a term, then the term is ground. The set of all ground terms that can be formed with the functions and constants of a language in L is called its Herbrand universe $U_{L}$.

An atomic formula (atom in short) is built from an n-ary predicate and *n* terms as its arguments, e.g., $p(t_{1},\ldots ,t_{n})$. An atom is ground if all terms $t_{i}$ are ground. The set of all ground atoms that can be formed in L is called Herbrand base $B_{L}$. Any atom and any negation of an atom is called a literal.

A rule of the following form is called a Horn clause:

$$A_{0}\leftarrow A_{1},\ldots ,A_{n}\;(n\geq 0),$$

where each $A_{i}$ (0≤i≤n) is an atom. $A_{0}$ is called the head of the clause, and the part on the right of ← is called its body. When i=0, the body of a rule is empty; such a rule, taking the form $A_{0}\leftarrow$, is called a fact. A classical logic program is a finite set of Horn clauses. Clauses (including facts) and logic programs containing no variables are called ground.

For any logic program *P*, we can define the language L(P) that consists of the predicates, functions, and constants occurring in *P*. If there are no constants in *P*, we add to L(P) a constant to avoid the empty domain. For simplicity, instead of $U_{L(P)}$ and $B_{L(P)}$, we will write $U_{P}$ and $B_{P}$, respectively. A Herbrand interpretation of *P* is any subset $I\subseteq B_{P}$ of its Herbrand base. Intuitively, the atoms in *I* are true, and all others are false. A Herbrand interpretation of *P* such that for each rule $A_{0}\leftarrow A_{1},\ldots ,A_{m}$ in *P*, this interpretation satisfies the logical formula $\forall X(\left(A_{1}\wedge \ldots \wedge A_{m}\right)\to A_{0})$ (*X* being the list of all variables occuring in the rule) is called the Herbrand model of *P*.

The notions of a Herbrand interpretation and Herbrand model can be generalized, in a natural way, to infinite sets of clauses. Let *P* be an arbitrary (finite or infinite) set of ground clauses. *P* defines an operator $T_{P}:2^{B_{P}}\mapsto 2^{B_{P}}$, where $2^{B_{P}}$ denotes the set of all Herbrand interpretations of *P*, by the following:

$$T_{P}\left(I\right)=\left\{A_{0}\in B_{P}|P\;\mathrm{contains}\;a\;\mathrm{rule}\;A_{0}\leftarrow A_{1},\ldots ,A_{m}\mathrm{such}\;\mathrm{that}\left\{A_{A},\ldots ,A_{m}\right\}\subseteq I\;\mathrm{holds}\right\}$$

This operator is called the immediate consequence operator; intuitively, it yields all atoms that can be derived by a single application of some rule in *P*, given the atoms in *I*. Since $T_{P}$ is monotone, by the Knaster–Tarski theorem, it has the least fixed point, denoted by $T_{P}^{\infty }$. It can be proven that $T_{P}^{\infty }$ is the limit of the sequence $T_{P}^{0}=\emptyset$, $T_{P}^{i+1}=T_{P}\left(T_{P}^{i}\right)$. A ground atom *A* is called a consequence of a set *P* of clauses if $A\in T_{P}^{\infty }$ (we write P⊧A). Additionally, we say that a negated ground atom ¬A is a consequence of *P* and write P⊧¬A if $A\notin T_{P}^{\infty }$.

The semantics of a set *P* of ground clauses, denoted as M(P), is defined as the following set consisting of atoms and negated atoms.

M(P)={A∣P⊧A}∪{¬A∣P⊧¬A}.

For a ground formula ϕ, built from literals with connective ∧,∨, we define P⊧ϕ if M(P)⊧ϕ according to the semantics of classical logic. The semantics of logic programs is now defined as follows. Let the grounding of a clause *r* in a language L, denoted as ground(r,L), be the set of all clauses obtained from *r* by all possible substitutions of elements of $U_{L}$ for the variables in *r*. For any logic program *P*, we define the following:

$$ground\left(P,L\right)=\underset{r\in P}{⋃}ground(r,L),$$

and we write ground(P) for ground(P,L(P)). The operator $T_{P}:2^{B_{P}}\mapsto 2^{B_{P}}$ associated with *P* is defined by $T_{P}=T_{ground(P)}$. Accordingly, M(P)=M(ground(P)).

### 2.2. Answer Set Programming

While a rule in classical logic programming is of the form $A_{0}\leftarrow A_{1},\ldots ,A_{m}$, in answer set programming a rule has the following form:

$$L_{1}\vee \ldots \vee L_{k}\vee ∼L_{k+1}\vee \ldots \vee ∼L_{l}\leftarrow L_{l+1}\wedge \ldots \wedge L_{m}\wedge ∼L_{m+1}\wedge \ldots \wedge ∼L_{n},$$

where all $L_{i}$, 1≤i≤n are literals (i.e., atoms or the negation of atoms) and 0≤k≤l≤m≤n. Here, ∼ is the default negation (negation as failure). For a rule *r* of the above form, $\left\{L_{1},\ldots ,∼L_{l}\right\}$ is the *head* of *r* and $\left\{L_{l+1},\ldots ,∼L_{n}\right\}$ is the body of *r*. We use Head(r) and Body(r) to denote the head and body of *r*, respectively.

The notion of an answer set is defined first for ground programs, which do not contain default negation. Let *P* be such a program and *M* be a consistent set of literals. We say that *M* is closed under *P* if for every rule r∈P, Head(r)∩M≠∅ whenever Body(r)⊆M. *M* is an answer set for *P* if *M* is minimal (relatively to set inclusion) among the sets of literals that are closed under *P*.

Now, we extend the definition of an answer set to ground programs with default negation. Let *P* be an arbitrary program and *M* a consistent set of literals. The reduct $P^{M}$ of *P* relative to *M* is the set of the following rule:

$$L_{1}\vee \ldots \vee L_{k}\leftarrow L_{l+1}\wedge \ldots \wedge L_{m}$$

for all rules $L_{1}\vee \ldots \vee L_{k}\vee ∼L_{k+1}\vee \ldots \vee ∼L_{l}\leftarrow L_{l+1}\wedge \ldots \wedge L_{m}\wedge ∼L_{m+1}\wedge \ldots \wedge ∼L_{n}$ in *P* such that *M* contains all literals $L_{k+1},\ldots ,L_{l}$ but does not contain any of $L_{m+1},\ldots ,L_{n}$. Thus, $P^{M}$ is a ground program without default negation. We say that *M* is an answer set for *P* if *M* is an answer set of $P^{M}$.

Finally, for a non-ground program *P*, the answer set for *P* is the answer set for ground(P). It can be verified that if a program *P* in answer set programming is also a classical logic program, then there is a unique answer set of *P*, and it coincides with the least fixed point of $T_{P}$. A ground literal *L* is entailed by a program *P*, denoted by P⊧L, if *L* is contained in all answer sets of *P*. For a ground formula ϕ built from literals with connective ∧,∨, P⊧ϕ is defined by interpreting ∧,∨ in the same way as in classical logic.

### 2.3. Transaction and Smart Contracts

Now, we are ready to define a new notion of transaction in which protection and certification are specified by logical programs and its semantics.

**Definition** **2**  (transaction). *A transaction T is a tuple (send,rece,sour,cert,prot), where send,rece,sour are defined in the same way as in LC, and the following:*

- *prot is the protection, which is a list of triples of logic programs, formulas and time locks. The number of triples must be the same as the number of receivers. Formally, $prot=\{\left(r_{1},\left(P_{1},\phi_{1},tilo_{1}\right)\right),\ldots ,\left(r_{m},\left(P_{m},\phi_{m},tilo_{m}\right)\right)\}$. $P_{i}$ is a logical program of answer set programming. $\phi_{i}$ is either the logical truth *⊤* or a non-ground formula ϕ built from literals with connective ∧,∨. $tilo_{i}$ is the time lock, which is of the form after(k) for some natural number k.*
- *cert is the certification, which is a set of ordered pairs of which the first component is the name of a transaction and the second component is either a set of literals or a valuation function that maps variables to constants. Formally, $cert=\{\left(T_{1},V_{1}\right),\ldots ,\left(T_{n},V_{n}\right)\}$.*
  *$V_{i}$ satisfies $\left(P_{i},⊤,tilo_{i}\right)$ if $V_{i}$ is an answer set for $P_{i}$ and the time lock is satisfied by the global clock. $V_{i}$ satisfies $\left(P_{i},\phi_{i},tilo_{i}\right)$, where $\phi_{i}$ is a non-ground formula ϕ built from literals with connective ∧,∨, if $P_{i}⊧V_{i}\left(\phi_{i}\right)$ and the time lock is satisfied by the global clock. When the time lock is ∅, it is vacuously satisfied by the global clock.*

Just like in LC, a transaction *T* redeems its sources if and only if the following holds:

1. The sender of *T* is one of the receivers in each of its source transactions.
2. The certification of *T* evaluates the protections of all its sources to be true.
3. None of its source transactions has been redeemed.

A transaction *T* is redeemed if one of its receivers has redeemed it.

We typically include a special predicate After(·) in our language. The atom After(t) is true when the global clock has passed time *t*. Some primitives of the post-quantum public key infrastructure [33] are also involved in our language. At the current stage, we do not specify which post-quantum algorithm is to be used because the standardization of post-quantum cryptography is still an ongoing procedure. Instead, we give an abstract description of some primitives of post-quantum cryptography in ASP. Those primitives include the encryption, signature and hash function.

- For encryption, a secret key sk is a constant. The function pk(·) maps sk to the corresponding public key pk(sk). The encryption function enc(·,·) maps a plain message *m* and public key pk=pk(sk) to an encrypted message enc(m,pk).
- For signature, the signing function sign(·,·) maps a message *m* and a secret key sk to the signature sign(m,sk). The atom verSig(σ,m,pk) is true when σ=sign(m,sk) is the signature of *m* and sk with public key pk=pk(sk).
- The hash function is represent by a collision-resistant function Hash.

Note that we use both an unconditionally secure signature and a post-quantum public key signature (e.g., lattice-based signature). They serve different purposes. The unconditionally secure signature is used to prove that a message from node *A* to other nodes is indeed sent from node *A*. Since the computer *A* may have more than one users, the post-quantum public key signature is used to prove the identity of the users of a computer.

This new format of transaction enables us to design various interesting smart contracts, especially contracts related to knowledge representation, automated planning, constraint solving and other areas in which ASP has proven to be applicable. Now, we use several examples for the demonstration.

**Figure 5:**
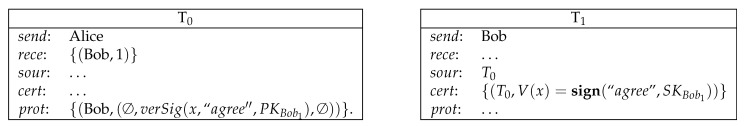
Authorized payment.

**Example** **5**  (authorized payment). *Bob receives a coin from Alice if he provides an appropriate signature from Bob${}_{1}$ (see Figure 5). Here, Bob is a (classical or quantum) computer, which is a node of the underlying quantum-secured permissioned blockchain. $Bob_{1}$ is a user of Bob. $PK_{Bob_{1}}$ and $SK_{Bob_{1}}$ are, respectively, the public and secret key of Bob${}_{1}$, and $\mathrm{sign}(“agree”,SK_{Bob_{1}})$ represents the signature of the message “agree” generated by secret key $SK_{Bob_{1}}$. Similarly, if Alice changes $verSig(x,“agree”,PK_{Bob_{1}})$ to $\left(verSig\left(x,“agree”,PK_{Bob_{1}}\right)\vee verSig\left(y,“agree”,PK_{Bob_{2}}\right)\right)$ $\wedge verSig(z,“agree”,PK_{Bob_{3}})$, then Bob has to provide a signature of Bob${}_{3}$ and at least one of the signatures of Bob${}_{1}$ and Bob${}_{2}$.*

**Figure 6:**
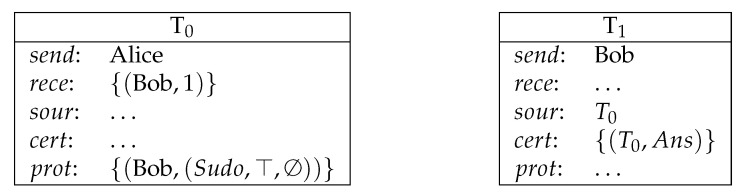
Conditional payment.

**Example** **6**  (conditional payment with Sudoku). *Bob receives 1 coin from Alice on the condition that Bob solves a Sudoku puzzle (see Figure 6). Here, Sudo is a Sudoku puzzle described in logical programs. A detailed formalization of Sudo can be found in Hölldobler and Schweizer [29]. Ans is an answer set for Sudo.*

Obviously, we can replace Sudo by any logical program. This means that we can realize various interesting conditional payments, as long as the condition can be expressed by logic programs.

**Figure 7:**
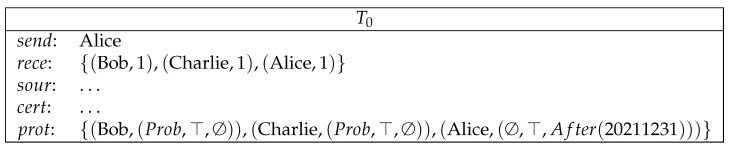
Competition for solving problems.

**Example** **7**  (competition for solving problems). *Alice declares a difficult problem Prob to Bob and Charlie. The one who first solves it before 20211231 will get the reward. (See Figure 7)*

#### 2.3.1. Multi-Party Lottery

Lottery is a part of the gambling industry with a turnover of billions of dollars [34]. Traditionally, a lottery game is organized by a trustworthy authority. In order to enter a lottery game, players buy tickets. Then, the authority organizing the game initiates a random process that determines the winning tickets. The revenue, in many cases, is large enough to induce temptation to cheat. To ensure fairness of a lottery game and trust of the players, several requirements on a lottery protocol were formulated (c.f. [16,35,36,37,38,39]):

1. Randomness. All tickets are equally likely to win.
2. Unpredictability. No player can predict the winning ticket.
3. Unforgeability. Tickets cannot be forged. In particular, it is impossible to create a winning ticket after the outcome of the random process is known.
4. Verifiability. The number and the revenue of winning tickets are publicly verifiable.
5. Decentralization. The random process does not rely on a single authority.

Note that decentralization allows to organize a lottery without the need for an authority that all players trust, replacing it by alternative mechanisms, such as Blockchain.

In [8], a two-party lottery protocol satisfying the above requirements is defined. We extend it to a multi-party lottery protocol.

**Example** **8**  (multi-party lottery). *The lottery protocol consists of several steps.*

**Figure 8:**
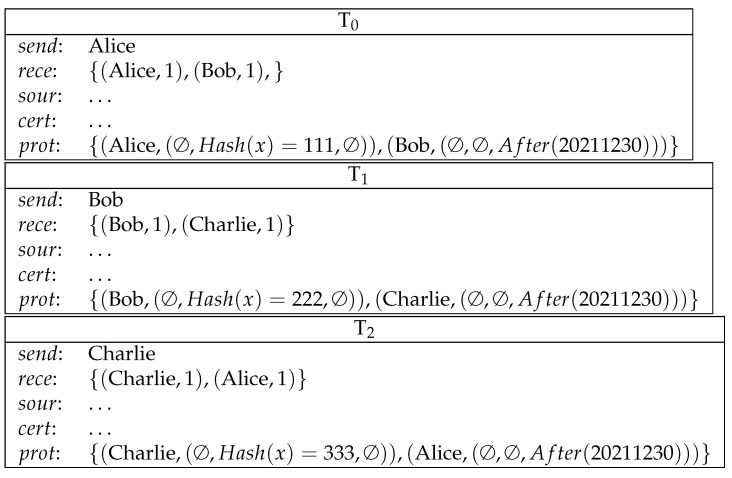
Lottery: deposit.

**Figure 9:**
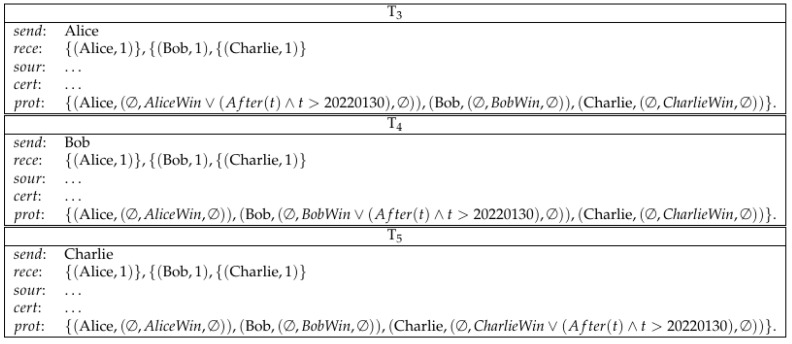
Lottery: conditional transfer.

**Figure 10:**
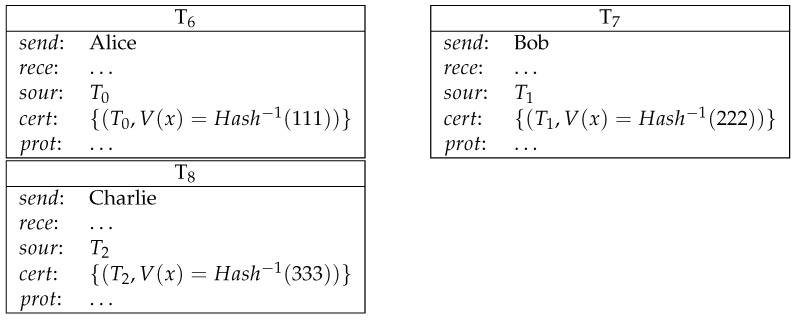
Lottery: revealing the secrets.

**Figure 11:**
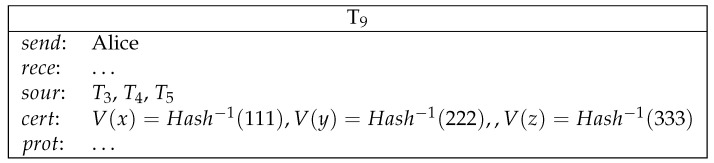
Lottery: determining the winner.

1. *Alice commits a secret to Bob by making a deposit. Bob commits a secret to Charlie by making a deposit. Charlie commits a secret to Alice by making a deposit. (See Figure 8).*
2. *Alice sends a conditional transfer to Alice, Bob and Charlie. Bob sends a conditional transfer to Alice, Bob and Charlie. Charlie sends a conditional transfer to Alice, Bob and Charlie. (See Figure 9).*
  *Here, AliceWin is specified by $\left(Hash^{-1}\left(x\right)=111\right)\wedge \left(Hash^{-1}\left(y\right)=222\right)\wedge \left(Hash^{-1}\left(z\right)=333\right)\wedge x+y+z\equiv_{3}0$, where $\equiv_{3}$ means equivalence modulo 3. BobWin is specified by $\left(Hash^{-1}\left(x\right)=111\right)\wedge \left(Hash^{-1}\left(y\right)=222\right)\wedge \left(Hash^{-1}\left(z\right)=333\right)\wedge x+y+z\equiv_{3}1$. CharlieWin is specified by $\left(Hash^{-1}\left(x\right)=111\right)\wedge \left(Hash^{-1}\left(y\right)=222\right)\wedge \left(Hash^{-1}\left(z\right)=333\right)\wedge x+y+z\equiv_{3}2$. Alice can also redeem her conditional transfer after 20220130. This condition ensures that Alice can get her money back in case any participant aborts the lottery game before the results can be determined. This is similarly the case for Bob and Charlie.*
3. *Alice reveals her secret and gets her deposit back. Bob reveals his secret and gets his deposit back. Charlie reveals his secret and gets his deposit back. (See Figure 10).*
4. *Now, Alice, Bob and Charlie’s secrets are public and the winner can be determined. The winner redeems the loser’s conditional transfer and his/her own conditional transfer. If Alice is the winner, then she redeems $T_{3}$, $T_{4}$ and $T_{5}$. This is similarly true for Bob/Charlie when they are the winner. (See Figure 11).*

#### 2.3.2. Legal Service

As in the last example, we show that our logical contracts can be used to provide a legal consultation service on blockchain. This is possible because ASP is able to express legal/normative rules, thanks to the close connection between ASP and default logic, which, in turn, can be used to express legal/normative rules. The connection between ASP and default logic is the following. Let *P* be a program such that the head of every rule of *P* is a single literal, as follows:

(1) $$L_{1}\leftarrow L_{2}\wedge \ldots \wedge L_{m}\wedge ∼L_{m+1}\wedge \ldots \wedge ∼L_{n}.$$

We can transform *P* into a default theory D(P) in the sense of [40] by turning each rule (1) into the default as follows:

$$\frac{L_{2}\wedge \ldots \wedge L_{m}\wedge :\neg L_{m+1},\ldots ,\neg L_{n}}{L_{1}}.$$

The correspondence between *P* and D(P) is the following: if *X* is an answer set for *P*, then the deductive closure of *X* is a consistent extension for D(P); conversely, every consistent extension for D(P) is the deductive closure of an answer set for *P*.

In deontic default logic [41], a conditional obligation ϕ→Oψ, meaning that if ϕ, then it is obliged to be ψ, and is expressed by a default as follows:

$$\frac{\phi :\psi }{\psi }.$$

Therefore, a conditional obligation $L_{1}\to OL_{2}$ can be expressed as the following rule in ASP:

$$L_{2}\leftarrow L_{1}\wedge ∼\neg L_{2}.$$

Moreover, since a conditional prohibition ϕ→Fψ, meaning that if ϕ, then it is forbidden to be ψ, is defined by ϕ→O¬ψ, we can express a conditional prohibition $L_{1}\to FL_{2}$ as the following rule in ASP:

$$\neg L_{2}\leftarrow L_{1}\wedge ∼L_{2}.$$

Now, if the protection of a transaction *T* includes a logical program *P*, which consists of some facts, conditional obligations and prohibitions expressed in ASP, then a transaction $T^{\prime }$ redeems *T* only if it provides an answer set of *P*. We can view *P* as a description of a legal situation or a normative system together with some facts. An answer set of *P* is then a suggestion of actions to be taken. In this sense, legal consultation can be carried out by transactions of blockchain.

## 3. Conclusions and Future Work

This paper applies answer set programming, enriched with post-quantum cryptographic primitives, to the design and specification of smart contracts on quantum-secured blockchains. The enrichment of post-quantum cryptographic primitives overcomes the limitation of unconditionally secure signatures in existing quantum-secured blockchains, which do not provide mechanisms for user authentication.

The application of ASP allows us to design various interesting smart contracts. Compared to procedural languages, the smart contracts written in our logical language are easier to be understood and formally verified.

In the future, we plan to systematically explore the usage of our framework in multi-party secure computation. We are also interested in further extending our logical language to include primitives of quantum computation. Such an extension will create a programming language of smart contracts in quantum logic.
